# Prediction of Breast Cancer Survival Using Clinical and Genetic Markers by Tumor Subtypes

**DOI:** 10.1371/journal.pone.0122413

**Published:** 2015-04-13

**Authors:** Nan Song, Ji-Yeob Choi, Hyuna Sung, Sujee Jeon, Seokang Chung, Sue K. Park, Wonshik Han, Jong Won Lee, Mi Kyung Kim, Ji-Young Lee, Keun-Young Yoo, Bok-Ghee Han, Sei-Hyun Ahn, Dong-Young Noh, Daehee Kang

**Affiliations:** 1 Cancer Research Institute, Seoul National University College of Medicine, Seoul, Korea; 2 Department of Biomedical Sciences, Seoul National University College of Medicine, Seoul, Korea; 3 Department of Preventive Medicine, Seoul National University College of Medicine, Seoul, Korea; 4 Division of Epidemiology and Genetics, National Cancer Institute, Rockville, Maryland, United States of America; 5 Department of Surgery, Seoul National University College of Medicine, Seoul, Korea; 6 Department of Surgery, University of Ulsan College of Medicine and ASAN Medical Center, Seoul, Korea; 7 Division of Cancer Epidemiology and Management, National Cancer Center, Goyang-si, Gyeonggi-do, Korea; 8 Cardiovascular Research Institute and Cardiovascular Genome Center, Yonsei University Health System, Seoul, Korea; 9 Center for Genome Science, Korea National Institute of Health, Osong, Korea; University of Texas MD Anderson Cancer Center, UNITED STATES

## Abstract

**Purpose:**

To identify the genetic variants associated with breast cancer survival, a genome-wide association study (GWAS) was conducted of Korean breast cancer patients.

**Methods:**

From the Seoul Breast Cancer Study (SEBCS), 3,226 patients with breast cancer (1,732 in the discovery and 1,494 in the replication set) were included in a two-stage GWAS on disease-free survival (DFS) by tumor subtypes based on hormone receptor (HR) and human epidermal growth factor receptor 2 (HER2). The associations of the re-classified combined prognostic markers through recursive partitioning analysis (RPA) of DFS for breast cancer were assessed with the Cox proportional hazard model. The prognostic predictive values of the clinical and genetic models were evaluated by Harrell’s C.

**Results:**

In the two-stage GWAS stratified by tumor subtypes, rs166870 and rs10825036 were consistently associated with DFS in the HR+ HER2- and HR- HER2- breast cancer subtypes, respectively (*P*_rs166870_=2.88×10^-7^ and *P*_rs10825036_=3.54×10^-7^ in the combined set). When patients were classified by the RPA in each subtype, genetic factors contributed significantly to differentiating the high risk group associated with DFS inbreast cancer, specifically the HR+ HER2- (*P*_discovery_=1.18×10^-8^ and *P*_replication_=2.08×10^-5^) and HR- HRE2- subtypes (*P*_discovery_=2.35×10^-4^ and *P*_replication_=2.60×10^-2^). The inclusion of the SNPs tended to improve the performance of the prognostic models consisting of age, TNM stage and tumor subtypes based on ER, PR, and HER2 status.

**Conclusion:**

Combined prognostic markers that include clinical and genetic factors by tumor subtypes could improve the prediction of survival in breast cancer.

## Introduction

Breast cancer is one of the most common malignancies among women in the world. Although breast cancer patients have generally a good prognosis[1], because the 5-year survival for invasive breast cancer cases from 1999 to 2005 was about 90%, large differences exist in survival rate because of a variety of clinicopathological prognostic factors[2]. The tumor-node-metastasis (TNM) staging system approved by the American Joint Committee on Cancer (AJCC) is a well-known important prognostic factor[3]. However, there are prognostic differences within specific stages because of the biological heterogeneity of tumors; thus, additional tumor markers such as tumor grade, lymphovascular invasion, proliferation markers, estrogen and progesterone receptor (ER and PR) status, and human epidermal growth factor receptor 2 (HER2) overexpression have been suggested to provide a more precise prognosis of breast cancer[3–5].

Among those prognostic factors, ER, PR, and HER2 status have been used for breast tumor subtypes classification in terms of heterogeneous clinical behavior and systematic therapy recommendations[6]. The tumor subtype based on ER, PR, and HER2 status has been validated in independent data set with significant differences in their clinical features even in Asian and European, early and metastatic breast cancer patients suggesting the robust classification[7–10].

In addition to clinicopathological prognostic factors, there is evidence supporting that inherited genetic factors influence the prognosis of breast cancer. Several genome-wide association studies (GWAS) have identified common variants associated with the prognosis of breast cancer at multiple genetic loci including *C10orf11*, *ARRDC3*, *RAD51L1*, *PBX1*, *RoR*_*α*_, *SYT6*, *NTN1*, *OCA2*, and *ZFHX3* genes[11–15]. Although genetic susceptibility markers influence differently the prognosis as well as the risk of breast cancer based on the ER, PR, and/or HER2 status[12,15–28], there are no genetic association studies on the prognosis of breast cancer which consider the heterogeneity of intrinsic tumor subtypes composed of various combinations of ER, PR, and HER2 status.

In this study, we hypothesized that the association of breast cancer prognosis with common genetic variants may vary by breast tumor subtypes. This study aims to conduct a two-stage GWAS for disease-free survival (DFS) in breast cancer stratified by tumor subtypes defined by the ER, PR, and HER2 status and evaluate the performance of prognostic models that included genetic variants with well-known clinical factors.

## Materials and Methods

### Study Population

The Seoul Breast Cancer Study (SEBCS) is a multicenter-based case-control study of female breast cancer in Seoul, Korea as previously reported[29,30]. This two-stage GWAS included a total of 3,226 incident breast cancer cases. A total of 4,040 histologically confirmed breast cancer patients were recruited from Seoul National University Hospital (SNUH) and ASAN Medical Center (AMC) between 2001 and 2007. For the discovery stage, 2,273 breast cancer patients who had been participated in GWAS on breast cancer risk were selected with sufficient DNA samples and successful genotyping[29]. We excluded subjects who had a previous history of breast or other cancers before the recruitment (N = 67), were diagnosed with benign breast disease (N = 17), or had no clinicopathological information (N = 73). After those exclusions which were not mutually exclusive, the subjects with a metastatic disease (N = 30) on review of their medical records were additionally excluded and 2,111 subjects remained. For survival analysis, the subjects who had a follow-up loss or follow-up time of less than 90 days (N = 113) were excluded and 1,998 subjects (95% of 2,111 eligible subjects) remained. Among those subjects, a total of 1,732 incident breast cancer patients with known tumor subtypes were included in the discovery set in this study.

For the replication set, a total of 1,837 breast cancer patients were included comprised of 508 SEBCS participants who were not included in the discovery set and 1,329 newly recruited participants who were histologically confirmed as having breast cancer at SNUH between 2000 and 2008. Of those patients, 1,735 breast cancer patients whose DNA samples were sufficient in concentration and purity were successfully genotyped. After exclusion in common with the discovery stage (N_previous history of cancers_ = 13, N_benign breast disease_ = 4, N_metastatic disease_ = 16, N_follow-up time of less than 90 days_ = 86), 1,616 subjects remained. The subjects with unknown tumor subtypes were also excluded, and a total of 1,494 subjects were included in the replication set in this study.

All participants in this study provided written informed consent. The study design was approved by the Committee on Human Research of Seoul National University Hospital (IRB No. H-0503-144-004).

### Tumor Subtypes

Information on ER, PR, and HER2 status was obtained from the medical records of patients’ based on laboratory results and the interpretation of pathologists. The ER and PR status was determined with immunohistochemistry (IHC) test. When ER and/or PR tumor cells showed 10% or more expression by IHC, the hormone receptor (HR) status was considered positive. Otherwise, HR was considered negative when both ER and PR tumor cells showed less than 10% expression by IHC. The HER2 status was defined by IHC and fluorescence in situ hybridization (FISH) tests according to HercepTest criteria[31]. When using the IHC staining score of HER2, 0 or 1+ was regarded as negative, while 3+ was considered as positive. When the IHC staining score of HER2 was 2+, the HER2 status was estimated with the FISH test. Tumor subtypes were classified as ER and/or PR positive and HER2 negative (HR+ HER2-), ER and/or PR positive and HER2 positive (HR+ HER2+), ER and PR negative and HER2 positive (HR- HER2+), and ER and PR negative and HER2 negative (HR- HER2-) subtypes.

### Genotyping and Quality Control

Genotyping was conducted using Affymetrix Genome-Wide Human SNP array 6.0 chip (Affymetrix, Inc.) and quality control steps ((a) a *p*-value<1.0×10^-6^ for deviation from Hardy-Weinberg equilibrium (HWE), (b) a call rate<95%, (c) a minor allele frequency (MAF)<1%, (d) a *p*-value<1.0×10^-4^ for differential missingness between cases and controls, and (e) multiple positioning and/or mitochondrial SNPs) were considered, as previously described[29]. Finally, a total of 555,525 genotyped SNPs remained in the discovery set. Moreover, an imputation of the SNPs based on the Han Chinese from Beijing and Japanese from Tokyo (CHB+JPT) data from the HapMap Phase II database (release 22) as a reference panel was done with the hidden Markov model using MaCH 1.0[32]. Among the 2,416,663 inferred SNPs, 2,210,580 remained after excluding SNPs that had an imputation quality score (r^2^) of <0.3 in the discovery set. When SNPs were genotyped as well as imputed, the information from the genotyped SNPs was used.

For the replication set, SNPs with a *p*-value less than 5.0×10^-6^ and a MAF equal to or more than 10% for the per allele hazard ratio (HR) were selected from each tumor subtype in the discovery stage. A total of 10 lead SNPs that included other SNPs in linkage disequilibrium (LD, r^2^>0.4) at loci with multiple SNPs were selected for genotyping in the replication stage as follows: rs161041, rs2835688, rs9935088, and rs166870 in HR+ HER2-; rs1896346 and rs12940572 in HR+ HER2+; rs34073156 and rs10906761 in HR- HER2+, and rs10825036 and rs10862597 in HR- HER2-. Proxy SNPs, rs1081228 (r^2^ = 0.98) and rs4750561 (r^2^ = 1.00), were genotyped for rs166870 at 15q25 and rs10906761 at 10p31, respectively, because of the genotyping failure of the original ones. The LD metrics (r^2^) of the selected SNP pairs were calculated using the SNP Annotation and Proxy Search (SNAP) based on HapMap release 22 in the CHB+JPT population panel. When the selected SNP pairs showed LD (r^2^>0.4), SNPs with the lowest *p*-value were selected for the per-allele HR, which were genotyped with the Fluidigm 192.24 Dynamic Array. Integrated Fluidic Circuit (IFC) (Fluidigm Corp. South San Francisco, CA, USA) was used according to the manufacturer’s instructions. When the selected SNPs failed genotyping, proxy SNPs were selected based on the LD metrics (r^2^) and genotyped. The success rates for genotyping were greater than 99% for all replication SNPs.

### Outcomes

The information on follow-up time, and recurrence status was obtained through retrospectively reviewing the patients’ medical records. The DFS time was defined as the time from the initial breast cancer surgery to the time of recurrence which includes loco-regional recurrence, first distant metastasis, contralateral breast cancer, and second primary cancer. The breast cancer patients who did not have evidence on recurrence were censored at last follow-up until 2011.

### Statistical Analysis

The associations between each SNP and DFS stratified by tumor subtypes were estimated with Cox proportional hazard models adjusted for age, recruiting center, and TNM stage. The hazard ratios (HRs) and 95% confidence intervals (CIs) per allele for each SNP were assessed in the additive model which was based on the number of rare alleles carried. The statistical significance of the associations was estimated with the *p*-value for the trend test with 1 degree of freedom. The analyses were done with the PLINK program version 1.07 and R 2.15.1 package (GenABEL and ProbABEL) and confirmed with SAS 9.3. To validate previously reported association, the SNPs identified from previous GWAS also analyzed. Using web-based Locus Zoom, regional association plots of the selected gene regions were generated. To estimate combined associations of the discovery and replication sets between SNPs and DFS, random-effects meta-analyses were done with STATA version 12.

A recursive partitioning analysis (RPA) of the prognostic factors was performed to classify breast cancer patients by distinguished groups based on the survival time[33]. The prognostic factors assessed by RPA were age, recruiting center, TNM stage, tumor subtype, and selected SNPs (rs166870 and rs10825036) were included. RPA was also done within specific tumor subtypes for those SNPs from the GWAS that were considered predictive factors. Kaplan-Meier graphs and HRs and 95% CIs of the Cox model are presented for the combined prognostic groups. Within each group, the probabilities of DFS and the percentage of breast cancer patients were measured. The predictive powers of survival models which included age, recruiting centers, TNM stage, and tumor subtypes with or without selected SNPs were calculated with Harrell’s C statistics, and the differences between the predictive powers were estimated with the *p*-value expressed by the lincom command in STATA. All statistical analyses were done again among patients with TNM stage I-III as a sensitivity analysis, and a statistically significant level was a two-sided *p*-value of 0.05.

## Results

### Characteristics of the Study Population

The characteristics of the 3,226 study participants and the associations with DFS are summarized in Table 1. The median follow-up time was 3.8 years (range, 0.3–8.0 years) in the discovery and 4.6 years (range, 0.3–8.5 years) in the replication sets. During the follow-up period, 214 (12.4%) patients in the discovery set and 164 (11.0%) patients in the replication set had events. Tumor size, nodal status, TNM stage, and tumor subtypes were statistically significant in associations with DFS in both the discovery and replication sets. The participants had a similar distribution for age, nodal status and ER and PR status, but a different distribution for tumor size, TNM stage, HER2 status, and breast tumor subtypes between discovery and replication sets (*p*-value<0.05 by Chi-square test). The characteristics including age, TNM stage, and tumor subtypes were not significantly different between remained and excluded subjects due to follow-up loss (data not shown). The characteristics of the study participants by tumor subtypes are presented in S1 Table.

**Table 1:** Characteristics of breast cancer patients and associations with disease-free survival (DFS).

	Discovery set						Replication set					
	N_total_	(%)	N_event_	(%)	HR^a^	(95% CI)	N_total_	(%)	N_event_	(%)	HR^a^	(95% CI)
No. of patients	1,732	(100.0)	214	(100.0)			1,494	(100.0)	164	(100.0)		
Median follow-up time, years (range)	3.8	(0.3–8.0)	2.1	(0.3–8.1)			4.6	(0.3–8.5)	2.2	(0.3–8.0)		
Total time at risk, person-years	6978.4		562.0				6677.8		429.4			
Median age at surgery, years (range)	47	(26–82)	46	(30–82)	1.01	(0.99–1.02)	47	(21–82)	44	(21–74)	0.98	(0.97–1.00)
Tumor size												
≤2 cm	942	(54.4)	69	(32.2)	1.00	ref.	798	(53.4)	54	(32.9)	1.00	ref.
2–5 cm	648	(37.4)	109	(50.9)	1.58	(1.15–2.18)	638	(42.7)	94	(57.3)	1.63	(1.15–2.32)
>5 cm	98	(5.7)	33	(15.4)	2.95	(1.87–4.64)	51	(3.4)	16	(9.8)	3.25	(1.79–5.87)
Nodal status												
negative	1,059	(61.1)	80	(37.4)	1.00	ref.	933	(62.5)	68	(41.5)	1.00	ref.
positive	673	(38.9)	134	(62.6)	2.15	(1.59–2.91)	561	(37.6)	96	(58.5)	2.19	(1.57–3.06)
TNM stage												
0	167	(9.6)	5	(2.3)	0.44	(0.18–1.12)	29	(1.9)	0	(0.0)	-	
I	596	(34.4)	43	(20.1)	1.00	ref.	566	(37.9)	35	(21.3)	1.00	ref.
II	694	(40.1)	87	(40.7)	1.51	(1.04–2.18)	705	(47.2)	82	(50.0)	1.85	(1.24–2.75)
III	275	(15.9)	79	(36.9)	4.15	(2.84–6.06)	194	(13.0)	47	(28.7)	4.63	(2.97–7.22)
ER status												
positive	1,069	(61.7)	100	(46.7)	1.00	ref.	921	(61.7)	81	(49.4)	1.00	ref.
negative	663	(38.3)	114	(53.3)	1.53	(0.13–2.08)	573	(38.4)	83	(50.6)	1.35	(0.96–1.89)
PR status												
positive	936	(54.0)	76	(35.5)	1.00	ref.	778	(52.1)	63	(38.4)	1.00	ref.
negative	793	(45.8)	138	(64.5)	1.75	(1.28–2.39)	714	(47.8)	101	(61.6)	1.33	(0.94–1.89)
HER2 status												
negative	1,270	(73.3)	152	(71.0)	1.00	ref.	1,186	(79.4)	107	(65.2)	1.00	ref.
positive	462	(26.7)	62	(29.0)	0.90	(0.66–1.23)	308	(20.6)	57	(34.8)	1.60	(1.15–2.24)
Tumor subtypes												
HR+ HER2-	995	(57.5)	91	(42.5)	1.00	ref.	907	(60.7)	68	(41.5)	1.00	ref.
HR+ HER2+	241	(13.9)	26	(12.2)	1.11	(0.72–1.73)	162	(10.8)	28	(17.1)	1.87	(1.20–2.93)
HR- HER2+	221	(12.8)	36	(16.8)	1.93	(1.30–2.86)	146	(9.8)	29	(17.7)	2.51	(1.61–3.90)
HR- HER2-	275	(15.9)	61	(28.5)	2.41	(1.73–3.35)	279	(18.7)	39	(23.8)	1.76	(1.18–2.62)

Abbreviations: DFS, disease-free survival; HR, hazard ratio; CI, confidence interval; ER, estrogen receptor; PR, progesterone receptor; HER2, human epidermal growth factor receptor 2; HR, hormone receptor; ref. reference.

^a^Cox proportional hazard model adjusted for age, recruiting center, TNM stage, and tumor subtypes.

### Genome-Wide Association Study on Prognosis

The associations between previously identified SNPs through the GWAS of prognosis and DFS in the SEBCS by tumor subtypes are listed in S2 Table. Although none of those SNPs reported in the previous GWAS were further replicated in the overall breast cancer, 4 SNPs showed significant associations with DFS in the specific tumor subtypes.

**Fig 1:**
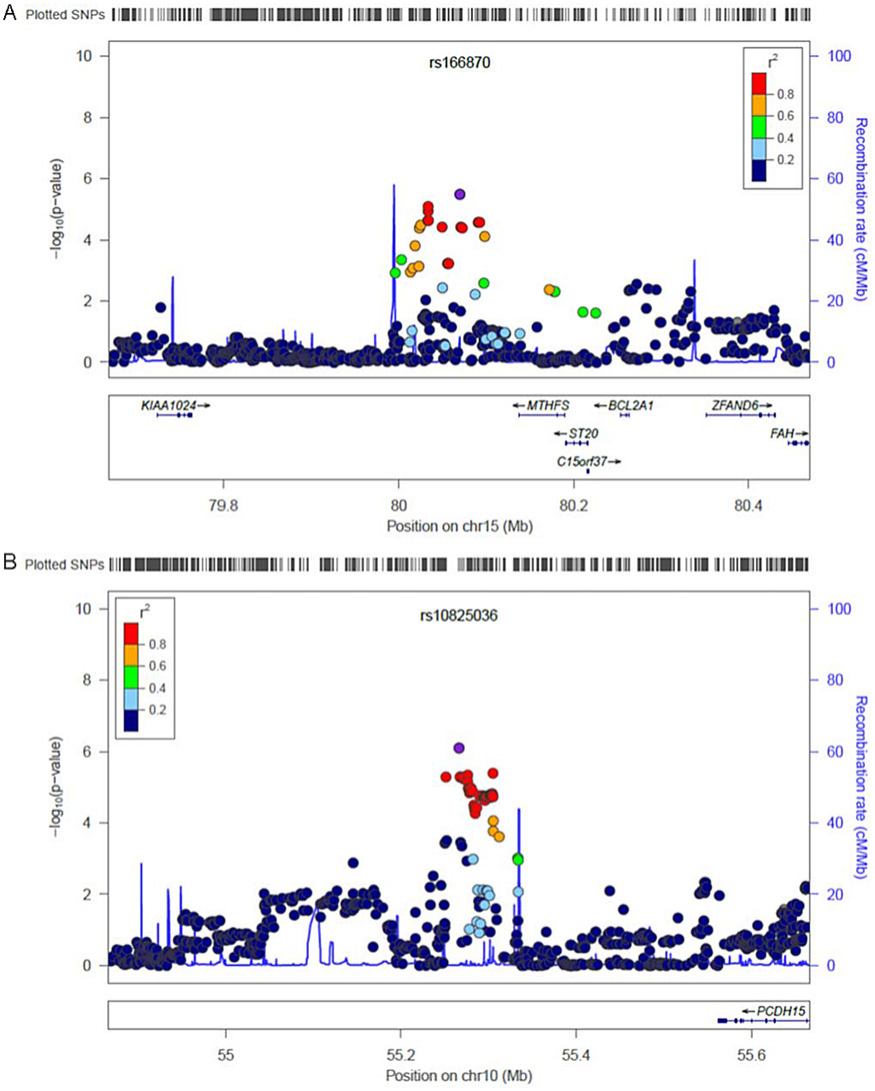
Regional plots for SNPs, (A) rs166870 and (B) rs10825036, in associations with DFS in the HR+ HER2- and HR- HER2- breast cancer subtype, respectively.

**Fig 2:**
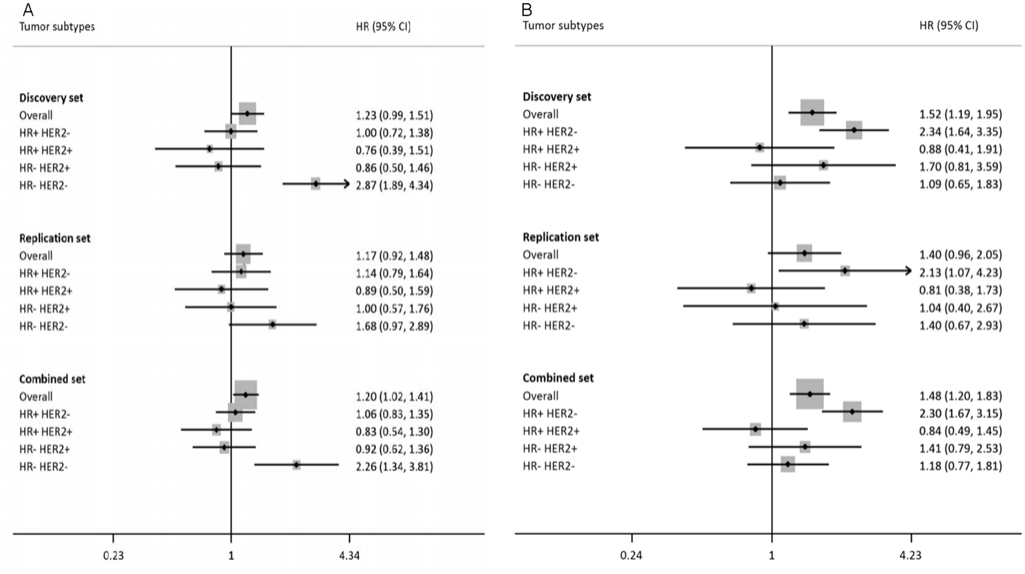
Associations between selected SNPs and disease-free survival (DFS) of breast cancer patients by tumor subtypes. (A) rs166870. (B) rs10825036.

Although there were no SNPs that reached a nominal genome-wide statistical significance (*p*-value<5.0×10^-8^), a total of 10 SNP for DFS achieved *p*-values of 5.0×10^-5f^ in each subtype in the discovery set (Table 2). Among these SNPs, rs166870 in HR+ HER2- (*p*_trend_ = 0.03) and rs10825036 in HR- HER2- (*p*_trend_ = 0.06) had statistically marginal significance in the replication set (Table 2). The regional plots for those two SNPs in associations with DFS in breast cancer for each subtype are shown in Fig 1. In combined analyses of the discovery and replication sets, those two SNPs had strong associations among breast cancer patients for each subtype (HR_rs166870_ = 2.30, 95% CI = 1.67–3.15, *p*_trend_ = 2.88×10^-7^ in HR+ HER2- and HR_rs10825036_ = 2.26, 95% CI = 1.34–3.81, *p*_trend_ = 3.54×10^-7^ in HR- HER2-, Table 2). The results were similar when breast cancer patients with TNM stage 0 were excluded (S3 Table). To identify the heterogeneity of the prognosis for those SNPs according to tumor subtypes, the associations with DFS for the other tumor subtypes of breast cancer were estimated, and they were not statistically associated with the other subtypes (Fig 2) and *p*-values for heterogeneity by tumor subtypes were statistically significant (*p*_rs166870_<0.01 and *p*_rs10825036_ = 0.02 in combined set).

**Table 2:** Associations between SNPs with the level of *p*-value<5.0×10^-6^ and disease-free survival (DFS) in breast cancer patients by tumor subtypes.

SNP	Loci	Nearby gene	Alleles^a^		Discovery set				Replication set				Combined set		
					MAF	HR^b^	(95% CI)	*P*	MAF^c^	HR^b^	(95% CI)	*P*	HR^c^	(95% CI)	*P*
**HR+ HER2-**															
rs161041	5q32	*PPP2R2B*	C	T	0.14	2.35	(1.68–3.30)	7.45×10^-7^	0.13	0.84	(0.50–1.43)	0.53	1.44	(0.53–3.93)	0.48
rs2835688	21q22	*intergenic/DSCR3*	A	G	0.26	2.65	(1.71–4.11)	1.40×10^-6^	0.27	1.07	(0.73–1.56)	0.74	1.67	(0.68–4.07)	0.26
rs9935088	16q23	*WWOX*	A	G	0.41	2.00	(1.50–2.66)	1.98×10^-6^	0.41	0.91	(0.64–1.29)	0.58	1.35	(0.62–2.94)	0.44
rs166870^d^	15q25	*intergenic/MTHFS*	C	T	0.13	2.34	(1.64–3.35)	3.16×10^-6^	0.87	2.13	(1.07–4.23)	0.03	2.30	(1.67–3.15)	2.88×10^-7^
**HR+ HER2+**															
rs1896346	12q24	*intergenic/TBX3*	G	A	0.38	0.12	(0.05–0.28)	1.09×10^-6^	0.44	1.08	(0.62–1.88)	0.80	0.37	(0.04–3.13)	0.36
rs12940572	17q21	*LASP1*	T	C	0.29	0.18	(0.09–0.36)	2.77×10^-6^	0.35	1.11	(0.63–1.96)	0.72	0.45	(0.07–2.72)	0.38
**HR- HER2+**															
rs34073156	8q24	*intergenic/LOC100129367*	G	A	0.10	4.14	(2.26–7.60)	4.58×10^-6^	0.07	1.27	(0.48–3.35)	0.64	2.44	(0.77–7.74)	0.13
rs10906761^e^	10p13	*intergenic/CDNF*	C	T	0.49	0.26	(0.14–0.47)	4.94×10^-6^	0.48	0.95	(0.54–1.68)	0.87	0.50	(0.14–1.80)	0.29
**HR- HER2-**															
rs10825036	10q21	*intergenic/PCDH15*	T	G	0.32	2.87	(1.90–4.34)	6.22×10^-7^	0.29	1.68	(0.97–2.89)	0.06	2.26	(1.34–3.81)	3.54×10^-7^
rs10862597	12q21	*intergenic/RPL6P25*	C	G	0.32	2.90	(1.76–4.77)	3.04×10^-6^	0.30	1.07	(0.67–1.70)	0.79	1.75	(0.66–4.66)	0.26

Abbreviations: SNP, single-nucleotide polymorphism; DFS, disease-free survival; RAF, risk-allele frequency; HR, hazard ratio; CI, confidence interval; MAF, minor allele frequency; HR, hormone receptor; HER2, human epidermal growth factor receptor 2.

^a^Major and minor alleles.

^b^Cox proportional hazard model adjusted for age, recruiting center, and TNM stage.

^c^Random-effect meta-analysis of discovery and replication set.

^d^A proxy SNP, rs1081228, was genotyped for rs166870 in the replication set (r2 = 0.96 and D' = 1.00 in CHB+JPT).

^e^A proxy SNP,rs4750561, was genotyped for rs10906761 in the replication set (r2 = 1.00 and D' = 1.00 in CHB+JPT).

### Prognostic Value of the Combined Markers of Clinical and Genetic Factors

RPA classified patients into distinct prognostic groups in each subtype shown in Table 3, which were significantly associated with the DFS of breast cancer in both the discovery and replication sets. The rs166870 (CC+CT or TT) was the second node among the HR+ HER2- patients after the TNM stage (0-II or III) (*p*_discovery_ = 1.18×10^-8^ and *p*_replication_ = 2.08×10–5, Table 2), and rs10825036 (TT+TG or GG) was the only node among the HR- HER2- patients showing significant differences between the groups (*p*_discovery_ = 2.35×10^-4^ and *p*_relication_ = 2.60×10^-2^, Table 3). The similar results were presented when breast cancer patients with TNM stage 0 were excluded (S4 Table).

**Table 3:** Associations between different combined groups of clinical and genetic factors and disease-free survival (DFS) among breast cancer patients.

	Discovery set			Replication set		
	HR^a^	(95% CI)	*P*	HR^a^	(95% CI)	*P*
Group by RPA among HR+ HER2- breast cancer patients						
Group 1: TNM stage 0-II and rs166870_CC+CT_	1.00	ref.	1.18×10^-8^	1.00	ref.	2.08×10^-5^
Group 2: TNM stage 0-II and rs166870_TT_	5.52	(2.00–15.28)		2.01	(0.90–4.47)	
Group 3: TNM stage III and rs166870_CC+CT_	3.61	(2.29–5.68)		3.07	(0.64–14.83)	
Group 4: TNM stage III and rs166870_TT_	10.50	(1.43–77.06)		7.26	(2.95–17.88)	
Group by RPA among HR- HER2- breast cancer patients						
Group1: rs10825036_TT+TG_	1.00	ref.	2.35×10^-4^	1.00	ref.	2.60×10^-2^
Group2: rs10825036_GG_	3.45	(1.78–6.67)		2.17	(1.10–4.28)	

Abbreviations: DFS, disease-free survival; HR, hazard ratio; CI, confidence interval; RPA, recursive partitioning analysis; HR, hormone receptor; HER2, human epidermal growth factor receptor 2.

^a^Cox proportional hazard model adjusted for age and recruiting center, additional TNM stage for group by tumor subtypes and selected SNPs.

The predictive powers of DFS for breast cancer were compared between the model with clinical variables alone and the model with combined clinical and genetic variables, and the latter tended to have better predictive powers in overall (Harrell’s C_clinical model_ = 70.92% and Harrell’s C_combined model_ = 71.37%, *p* = 0.03), HR+ HER2- (Harrell’s C_clinical model_ = 65.08% and Harrell’s C_combined model_ = 66.69%, *p*<0.01), and HR- HER2- breast cancer (Harrell’s C_clinical model_ = 63.26% and Harrell’s C_combined model_ = 65.88%, *p*<0.01).

## Discussion

From the two-stage GWAS, genetic factors that were associated with DFS in breast cancer were identified by tumor subtypes, and the prognostic values for the combined clinical and genetic factors were evaluated. The SNPs, rs166870 and rs10825036, showed a statistically significant association with DFS in the HR+ HER2- and HR- HER2-breast tumor subtypes, respectively, and these associations were not seen in the other tumor subtypes. They contributed to the prognostic models by improving the prediction of DFS within specific subtypes.

We conducted a subtype-specific GWAS, unlike other previous studies that had conducted a GWAS for overall breast cancer before stratifying by ER, PR, and HER2 status because breast cancer is considered as a heterogeneous disease for which the prognosis varies across subtypes[34]. This intertumor heterogeneity is plausible in that breast cancer could originate from different cell types according to the tumor subtype[35] and is supported by previous studies showing the heterogeneous associations between SNPs and the prognosis of breast cancer by ER, PR and HER2 status[15,18–27], in agreement with the current study (Fig 2). Another reason for the subtype-specific analyses was that breast cancer subtypes are considered as a predictor factor that distinguishes different responses to particular therapies among patients[36]. Because those differences in responses to particular therapies could have been a result of subtype-specific biological differences, the stratification of breast tumors by subtypes is necessary[37].

Among previously identified SNPs by GWAS for the prognosis of breast cancer, none of the SNPs were associated with DFS overall in this study (S2 Table). Of those SNPs that showed an association in the subtypes, rs3784099 and rs9934948 had been associated with the total mortality, for overall and ER+ breast cancer in Chinese women[15]. Although the association of SNP rs9934948 was not in the same direction as in this study, the reason for this might be because the tumor subtypes, specifically HR+ and HR-, had a different tumor biology from that of ErbB2, and the luminal subtypes showed entirely different up-regulated gene patterns even in the same organ relapse patients[38]. The other identified SNPs, rs1387389, rs2774307, and rs4778137 (especially in ER-), are associated with survival in European women, and the same directions for the estimates are shown in our patients[12,14]. The SNP rs4778137 is also significantly associated with the overall survival (OS) of breast cancer in Chinese women, even though it has not been replicated in the ER- subtype[15].

In the region surrounding the SNP rs166870, an acetylation of lysine 27 as an activation mark in the H3 histone protein (H3K27Ac) was observed by *in silico* analysis (S1A Fig). Rs166870 is close to the methenyltetrahydrofolate synthetase (*MTHFS*) gene, which is involved in folate mediated one-carbon metabolism. Although associations between SNPs in the *MTHFS* gene and the risk and prognosis of breast cancer have not been reported, an association has been reported between *MTHFS* variants and the prognosis of lung cancer[39], and also other one-carbon metabolism pathway genes are associated with the prognosis of breast cancer[22,40–42]. One-carbon metabolism influences DNA methylation and synthesis[43], regulating Bcl-2/adenovirus E1B 19 kDa-interacting protein 3 (BNIP3). The loss of BNIP3 expression has been correlated with poor prognostic features such as lymph node metastasis, a higher mitotic activity index (MAI), and tubule formation in breast cancer[44]. Moreover, the MTHFS protein is known as a potential mediator of insulin-like growth factor-1 receptor (IGF-1R) dependent transformation[45]. Breast cancer patients, especially HR+, HER2-, and tumor patients with a Ki-67≥14%, who had a better score for IGF-1R expression had a higher survival[46].

SNP rs10825036 was also represented as a H3K27Ac mark by *in silico* analysis (S1B Fig). Although there are no studies on rs10825036, the SNP was weakly correlated with rs583012, which was associated with the c-reactive protein[47], and rs12256830 was associated with antibody levels[48]. Rs10825036 is close to the *PCDH15* gene which encodes integral membrane proteins that mediate calcium-dependent cell-cell adhesion. Previously, the SNPs of the *PCDH15* gene are known for associations with adverse events caused by chemotherapy in breast cancer[49] as well as with lipid abnormalities[50]. The lipid profiles have been associated with the risk, stage, and recurrence of breast cancer[51–53]. Moreover, lipids profiles have been distinguished between triple-negative and other breast tumor subtypes[54].

The predictive power of the combined model including rs166870 and rs10825036 identified from the two-stage GWAS, was more improved than that of the clinical model which did not include the SNPs. In previous multivariate survival models, Harrell’s C statistics were estimated ranging from 0.69 to 0.82 according to the number and type of clinicopathological factors and the characteristics of the study population included in the models[55–57]. There were no SNPs whose c-indices were estimated, but the gene expression signatures improved the predictive powers when additionally included in multivariate clinicopathological models[58].

To assign the risk group according to the prognosis of breast cancer, clinical and genetic factors were combined and re-classified with RPA. From the results of the RPA, the genetic factors selected from the two-stage GWAS were more valuable when the analyses were stratified by tumor subtypes, and only one node of the genetic factors was statistically significant regardless of the clinical factors in HR- HER2- breast cancer. Therefore, prognostic markers that include the SNPs identified from the GWAS could be valuable in predicting the prognosis of breast cancer, particularly in specific tumor subtypes.

This is the first study that conducted a two-stage GWAS by tumor subtypes based on HR and HER2 status. Furthermore, combined survival models that include genetic factors identified by the two-stage GWAS as well as other well-known clinical factors were evaluated for predicting the prognosis of breast cancer. The first limitation of this study was that the statistical significances of the associations from the two-stage GWAS did not reach a *p*-value<5.0×10^-8^ as the nominal significance for the GWAS[59]. However, there have been a few GWAS on the prognosis of breast cancer, and none of the SNPs associated with the prognosis of breast cancer have had a nominal significance from the GWAS so far[11–15]. Second, the treatment information for breast cancer was not controlled in the analyses because of substantial missing data. Although the adjuvant chemotherapy and radiation did not affect associations of survival, the hormone therapy was associated with survival in the discovery set but not in the replication set (data not shown), which tended to depend on the tumor subtypes. All the analyses were adjusted or stratified by tumor subtypes instead of controlling for treatments.

It has been inconclusive whether genetic factors influence survival by intrinsic subtypes. In this analysis, the novel genetic markers including rs166870 and rs10825036 were associated with survival in HR+ HER2- and HR- HER2- tumors showing heterogeneity between tumor subtypes. The novel genetic markers identified in this study would be helpful to understand biological insights in heterogeneous breast cancer patients. Furthermore, RPA showed those genetic markers played a role in distinguishing between high and low risk groups of breast cancer patients. The combined prognostic markers that include the genetic markers and well-known clinical factors could be useful to predict the clinical outcome for breast cancer patients.

In conclusion, our two-stage GWAS identified two novel SNPs (rs166870 and rs10825036) associated with DFS in the HR+ HER2- and HR- HER2- subtypes, respectively. When these genetic factors were added to well-known clinical survival models that included age, TNM stage, and tumor subtype, improved predictive powers of the models were observed. Furthermore, our RPA showed that genetic factors had a role in distinguishing between high and low risk groups when using combined prognostic markers. To validate these results, further studies are needed to evaluate the predictive power of the survival models which include genetic factors as well as clinical factors.

## Supporting Information

S1 Fig *In silico* analysis of the region surrounding the selected SNPs. (A) rs166870 and (B) rs10825036.
(TIF)

S1 Table Characteristics of breast cancer patients by tumor subtypes. (DOCX)

S2 Table Associations between previously identified SNPs and DFS of breast cancer by tumor subtypes in the discovery set. (DOCX)

S3 Table Sensitivity analysis on associations between selected SNPs and disease-free survival (DFS) in breast cancer patients by tumor subtypes with stage I-III. (DOCX)

S4 Table Sensitivity analysis on associations between different combined groups of clinical and genetic factors and disease-free survival (DFS) in breast cancer patients subtypes with stage I-III. (DOCX)
