# Supplementary figures and images for: Prediction of Breast Cancer Survival Using Clinical and Genetic Markers by Tumor Subtypes
Source: PLoS One. 2015 Apr 13;10(4):e0122413. doi: 10.1371/journal.pone.0122413 (PMC4395109; doi:10.1371/journal.pone.0122413)

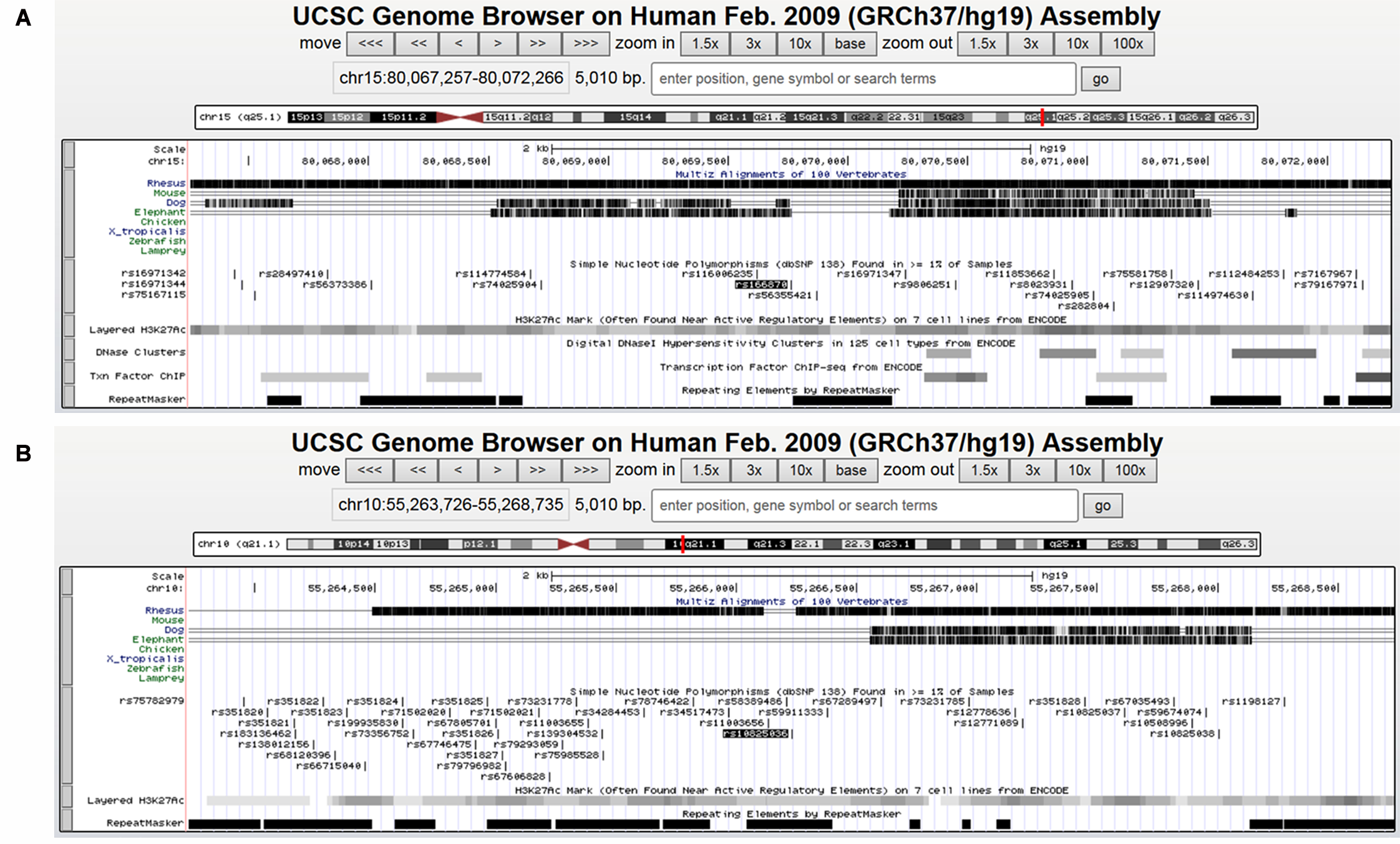

Supplement: S1 Fig — (A) rs166870 and (B) rs10825036. (TIF) [file pone.0122413.s001.tif]
