# Supplementary material for: Prediction of Breast Cancer Survival Using Clinical and Genetic Markers by Tumor Subtypes
Source: PLoS One. 2015 Apr 13;10(4):e0122413. doi: 10.1371/journal.pone.0122413 (PMC4395109; doi:10.1371/journal.pone.0122413)
Supplement: S1 Table — (DOCX) [file pone.0122413.s002.docx]

| S1 Table. Characteristics of breast cancer patients by tumor subtypes | | | | | | | | | | | | | | | | |  |
| --- | --- | --- | --- | --- | --- | --- | --- | --- | --- | --- | --- | --- | --- | --- | --- | --- | --- |
|  | HR+ HER2- | | | | HR+ HER2+ | | | | HR- HER2+ | | | | HR- HER2- | | | | |
|  | Discovery | | Replication | | Discovery | | Replication | | Discovery | | Replication | | Discovery | | Replication | | |
|  | N | (%) | N | (%) | N | (%) | N | (%) | N | (%) | N | (%) | N | (%) | N | (%) | |
| No. of patients | 995 | (100.0) | 907 | (100.0) | 241 | (100.0) | 162 | (100.0) | 221 | (100.0) | 146 | (100.0) | 275 | (100.0) | 279 | (100.0) | |
| Median follow-up time, years (range) | 4.0 | (0.3-8.8) | 4.6 | (0.3-8.5) | (3.5) | (0.5-8.5) | 4.8 | (0.3-8.4) | 3.5 | (0.4-8.4) | 4.5 | (0.3-8.5) | 3.5 | (0.3-8.5) | 4.3 | (0.3-8.5) | |
| Total time at risk, person-years | 4139.3 | | 4137.9 | | 972.3 | | 756.5 | | 841.4 | | 632.7 | | 1025.3 | | 1150.7 | | |
| Recurrence | 91 | (9.2) | 68 | (7.5) | 26 | (10.8) | 28 | (17.3) | 36 | (16.3) | (29.0) | (19.9) | 61 | (22.2) | 39 | (14.0) | |
| Death | 37 | (3.7) | 22 | (2.4) | 15 | (6.2) | 11 | (6.8) | 20 | (9.1) | (14.0) | (9.6) | 33 | (12.0) | 25 | (9.0) | |
| Median age at surgery, years (range) | 47 | (26-80) | 47 | (21-82) | 46 | (28-70) | 44 | (25-65) | 49 | (30-79) | (50.0) | (28-82) | 46 | (26-82) | 46 | (21-78) | |
| Tumor size |  |  |  |  |  |  |  |  |  |  |  |  |  |  |  |  | |
| ≤2 cm | 581 | (58.4) | 544 | (60.0) | 132 | (54.8) | 77 | (47.5) | 124 | (56.1) | (65.0) | (44.5) | 105 | (38.2) | 445 | (40.1) | |
| 2-5 cm | 345 | (34.7) | 335 | (36.9) | 82 | (34.0) | 75 | (46.3) | 76 | (34.4) | (76.0) | (52.1) | 145 | (52.7) | 485 | (54.5) | |
| >5 cm | 40 | (4.0) | 23 | (2.5) | 19 | (7.9) | 9 | (5.6) | 17 | (7.7) | (5.0) | (3.4) | 22 | (8.0) | 47 | (5.0) | |
| Nodal status |  |  |  |  |  |  |  |  |  |  |  |  |  |  |  |  | |
| negative | 624 | (62.7) | 572 | (63.1) | 144 | (59.8) | 88 | (54.3) | 136 | (61.5) | (92.0) | (63.0) | 155 | (45.4) | 181 | (64.9) | |
| positive | 371 | (37.3) | 335 | (36.9) | 97 | (40.3) | 74 | (45.7) | 85 | (38.5) | (54.0) | (37.0) | 120 | (43.6) | 98 | (35.1) | |
| TNM stage |  |  |  |  |  |  |  |  |  |  |  |  |  |  |  |  | |
| 0-I | 482 | (48.4) | 403 | (44.4) | 107 | (44.4) | 56 | (34.6) | 98 | (44.3) | (47.0) | (32.2) | 76 | (27.6) | 89 | (31.9) | |
| II | 384 | (38.6) | 404 | (44.5) | 88 | (36.5) | 80 | (49.4) | 77 | (34.8) | (80.0) | (54.8) | 145 | (52.7) | 141 | (50.5) | |
| III | 129 | (13.0) | 100 | (11.0) | 46 | (19.1) | 26 | (16.1) | 46 | (20.8) | (19.0) | (13.0) | 54 | (19.6) | 49 | (17.6) | |
| ER status |  |  |  |  |  |  |  |  |  |  |  |  |  |  |  |  | |
| positive | 880 | (88.4) | 794 | (87.5) | 189 | (78.4) | 127 | (78.4) | 0 | (0.0) | (0.0) | (0.0) | 0 | (0.0) | 0 | (0.0) | |
| negative | 115 | (11.6) | 113 | (12.5) | 52 | (21.6) | 35 | (21.6) | 221 | (100.0) | (146.0) | (100.0) | 275 | (100.0) | 279 | (100.0) | |
| PR status |  |  |  |  |  |  |  |  |  |  |  |  |  |  |  |  | |
| positive | 751 | (75.5) | 670 | (73.9) | 185 | (76.8) | 108 | (66.7) | 0 | (0.0) | (0.0) | (0.0) | 0 | (0.0) | 0 | (0.0) | |
| negative | 241 | (24.2) | 236 | (26.0) | 56 | (23.2) | 53 | (32.7) | 221 | (100.0) | (146.0) | (100.0) | 275 | (100.0) | 279 | (100.0) | |
| HER2 status |  |  |  |  |  |  |  |  |  |  |  |  |  |  |  |  | |
| negative | 0 | (0.0) | 0 | (0.0) | 241 | (100.0) | 162 | (100.0) | 221 | (100.0) | (146.0) | (100.0) | 0 | (0.0) | 0 | (0.0) | |
| positive | 995 | (100.0) | 907 | (100.0) | 0 | (0.0) | 0 | (0.0) | 0 | (0.0) | (0.0) | (0.0) | 275 | (100.0) | 279 | (100.0) | |

Abbreviations: HR, hormone receptor; HER2, human epidermal growth factor receptor 2; ER, estrogen receptor; PR, progesterone receptor.
