# Supplementary material for: Prediction of Breast Cancer Survival Using Clinical and Genetic Markers by Tumor Subtypes
Source: PLoS One. 2015 Apr 13;10(4):e0122413. doi: 10.1371/journal.pone.0122413 (PMC4395109; doi:10.1371/journal.pone.0122413)
Supplement: S2 Table — (DOCX) [file pone.0122413.s003.docx]

| S2 Table. Associations between previously identified SNPs and DFS of breast cancer by tumor subtypes in the discovery set | | | | | | | | | | | | | | | | |
| --- | --- | --- | --- | --- | --- | --- | --- | --- | --- | --- | --- | --- | --- | --- | --- | --- |
| SNP^a^ | Loci | Gene | Alleles^b^ | | SEBCS | | | | | | Previous GWAS | | | | | |
|  |  |  |  |  | MAF | Subtype | HR^c^ | (95% CI) | *P*_trend_ | Genotyping | MAF | Outcome | HR | (95% CI) | *P*_trend_ | Ref. |
| rs10509373 | 10q22 | *C10orf11* | T | C | 0.03 | Overall | 1.78 | (0.86-3.69) | 0.12 | imputed | 0.02 | RFS | 4.53 | (2.62-7.83) | 6.3×10^-8^ | ([1](#_ENREF_1)) |
|  |  |  |  |  |  | HR+ HER2- | 3.53 | (0.82-15.32) | 0.09 |  |  |  |  |  |  |  |
|  |  |  |  |  |  | HR+ HER2+ | 0.50 | (0.12-2.14) | 0.35 |  |  |  |  |  |  |  |
|  |  |  |  |  |  | HR- HER2+ | 0.71 | (0.09-5.75) | 0.75 |  |  |  |  |  |  |  |
|  |  |  |  |  |  | HR- HER2- | 0.78 | (0.22-2.76) | 0.70 |  |  |  |  |  |  |  |
| rs421379 | 5q14 | *ARRDC3* | C | T | 0.03 | Overall | 0.91 | (0.51-1.64) | 0.76 | typed | 0.05 | BCSS | 1.61 | (1.33-1.96) | 9.5×10^-7^ | ([2](#_ENREF_2)) |
|  |  |  |  |  |  | HR+ HER2- | 0.95 | (0.39-2.30) | 0.90 |  |  |  |  |  |  |  |
|  |  |  |  |  |  | HR+ HER2+ | 1.66 | (0.58-4.74) | 0.34 |  |  |  |  |  |  |  |
|  |  |  |  |  |  | HR- HER2+ | 1.19 | (0.17-8.09) | 0.86 |  |  |  |  |  |  |  |
|  |  |  |  |  |  | HR- HER2- | 0.24 | (0.03-1.77) | 0.16 |  |  |  |  |  |  |  |
| rs3784099 | 14q24 | *RAD51L1* | G | A | 0.15 | Overall | 1.27 | (0.96-1.67) | 0.09 | imputed | 0.13 | DFS OS | 1.43 1.49 | (1.25-1.64) (1.28-1.72) | 2.8×10^-7^ 1.2×10^-7^ | ([3](#_ENREF_3)) |
|  |  |  |  |  |  | HR+ HER2- | 1.51 | (0.94-2.44) | 0.09 |  |  |  |  |  |  |  |
|  |  |  |  |  |  | HR+ HER2+ | **3.77** | **(1.12-12.74)** | **0.03** |  |  |  |  |  |  |  |
|  |  |  |  |  |  | HR- HER2+ | 0.92 | (0.48-1.79) | 0.81 |  |  |  |  |  |  |  |
|  |  |  |  |  |  | HR- HER2- | 1.14 | (0.71-1.84) | 0.59 |  |  |  |  |  |  |  |
| rs1387389 | 1q23 | *PBX1* | A | G | 0.49 | Overall | 1.15 | (0.95-1.38) | 0.15 | imputed | 0.36 | BCSS | 1.28 | (1.16-1.43) | 3.8×10^-6^ | ([2](#_ENREF_2)) |
|  |  |  |  |  |  | HR+ HER2- | 1.26 | (0.94-1.71) | 0.13 |  |  |  |  |  |  |  |
|  |  |  |  |  |  | HR+ HER2+ | 1.03 | (0.57-1.88) | 0.92 |  |  |  |  |  |  |  |
|  |  |  |  |  |  | HR- HER2+ | **1.73** | **(1.05-2.85)** | **0.03** |  |  |  |  |  |  |  |
|  |  |  |  |  |  | HR- HER2- | 0.96 | (0.67-1.38) | 0.83 |  |  |  |  |  |  |  |
| rs3884558 | 15q22 | *RORα* | A | G | 0.31 | Overall | 1.04 | (0.86-1.27) | 0.69 | typed | 0.07 | BCSS | 1.46 | (1.24-1.72) | 3.9×10^-6^ | ([2](#_ENREF_2)) |
|  |  |  |  |  |  | HR+ HER2- | 0.96 | (0.70-1.32) | 0.81 |  |  |  |  |  |  |  |
|  |  |  |  |  |  | HR+ HER2+ | 1.15 | (0.66-2.03) | 0.62 |  |  |  |  |  |  |  |
|  |  |  |  |  |  | HR- HER2+ | 0.89 | (0.51-1.53) | 0.66 |  |  |  |  |  |  |  |
|  |  |  |  |  |  | HR- HER2- | 1.09 | (0.73-1.64) | 0.66 |  |  |  |  |  |  |  |
| rs2774307 | 1p13 | *SYT6* | G | A | 0.08 | Overall | 0.96 | (0.69-1.32) | 0.78 | typed | 0.26 | BCSS | 1.30 | (1.16-1.47) | 7.9×10^-6^ | ([2](#_ENREF_2)) |
|  |  |  |  |  |  | HR+ HER2- | 0.63 | (0.33-1.22) | 0.17 |  |  |  |  |  |  |  |
|  |  |  |  |  |  | HR+ HER2+ | 1.09 | (0.42-2.85) | 0.87 |  |  |  |  |  |  |  |
|  |  |  |  |  |  | HR- HER2+ | 1.13 | (0.48-2.69) | 0.78 |  |  |  |  |  |  |  |
|  |  |  |  |  |  | HR- HER2- | 1.60 | (0.92-2.77) | 0.10 |  |  |  |  |  |  |  |
| rs3785982 | 17p13 | *NTN1* | C | T | 0.19 | Overall | 1.03 | (0.82-1.28) | 0.82 | typed | 0.12 | BCSS | 1.40 | (1.21-1.62) | 7.9×10^-6^ | ([2](#_ENREF_2)) |
|  |  |  |  |  |  | HR+ HER2- | 1.12 | (0.77-1.62) | 0.55 |  |  |  |  |  |  |  |
|  |  |  |  |  |  | HR+ HER2+ | 1.24 | (0.69-2.24) | 0.47 |  |  |  |  |  |  |  |
|  |  |  |  |  |  | HR- HER2+ | 0.54 | (0.28-1.04) | 0.06 |  |  |  |  |  |  |  |
|  |  |  |  |  |  | HR- HER2- | 0.88 | (0.55-1.39) | 0.57 |  |  |  |  |  |  |  |
| rs4778137 | 15q13 | *OCA2* | C | G | 0.22 | Overall | 0.95 | (0.74-1.22) | 0.69 | imputed | 0.30 | OS OS, ER+ OS, ER- | 0.93 0.99 0.82 | (0.87-0.98) (0.91-1.10) (0.73-0.92) | 0.01 0.75 5.0×10^-4^ | ([4](#_ENREF_4)) |
|  |  |  |  |  |  | HR+ HER2- | 1.42 | (0.98-2.06) | 0.07 |  |  |  |  |  |  |  |
|  |  |  |  |  |  | HR+ HER2+ | 0.63 | (0.26-1.51) | 0.63 |  |  |  |  |  |  |  |
|  |  |  |  |  |  | HR- HER2+ | 1.40 | (0.76-2.56) | 0.28 |  |  |  |  |  |  |  |
|  |  |  |  |  |  | HR- HER2- | **0.45** | **(0.25-0.84)** | **0.01** |  |  |  |  |  |  |  |
| rs9934948 | 16q22 | *ZFHX3* | T | C | 0.38 | Overall | 0.90 | (0.73-1.11) | 0.30 | imputed | 0.46 | DFS OS | 1.19 1.29 | (1.08-1.31) (1.16-1.44) | 7.3×10^-4^ 5.8×10^-4^ | ([3](#_ENREF_3)) |
|  |  |  |  |  |  | HR+ HER2- | 0.87 | (0.61-1.23) | 0.42 |  |  |  |  |  |  |  |
|  |  |  |  |  |  | HR+ HER2+ | 2.00 | (0.91-4.41) | 0.09 |  |  |  |  |  |  |  |
|  |  |  |  |  |  | HR- HER2+ | **0.54** | **(0.32-0.90)** | **0.02** |  |  |  |  |  |  |  |
|  |  |  |  |  |  | HR- HER2- | 1.08 | (0.73-1.60) | 0.70 |  |  |  |  |  |  |  |
| Abbreviations: SNP, single-nucleotide polymorphism; DFS, disease-free survival; SEBCS, Seoul breast cancer study; GWAS, genome-wide association study; MAF, minor allele frequency; HR, hazard ratio; CI, confidence interval; Ref. reference; HR, hormone receptor; HER2, human epidermal growth factor receptor 2; RFS, recurrence-free survival; BCSS, breast cancer-specific survival; OS, overall survival. | | | | | | | | | | | | | | | | |
| ^a^SNP identified by previous GWAS on survival. | | | | | | | | | | | | | | | | |
| ^b^Major and minor alleles. | | | | | | | | | | | | | | | | |
| ^c^Cox proportional hazard model adjusted for age, recruiting center, and TNM stage. | | | | | | | | | | | | | | | | |

**References**

1. Kiyotani K, Mushiroda T, Tsunoda T, Morizono T, Hosono N, Kubo M, et al. A genome-wide association study identifies locus at 10q22 associated with clinical outcomes of adjuvant tamoxifen therapy for breast cancer patients in Japanese. Human molecular genetics. 2012;21:1665-72.

2. Rafiq S, Tapper W, Collins A, Khan S, Politopoulos I, Gerty S, et al. Identification of inherited genetic variations influencing prognosis in early-onset breast cancer. Cancer research. 2013;73:1883-91.

3. Shu XO, Long J, Lu W, Li C, Chen WY, Delahanty R, et al. Novel genetic markers of breast cancer survival identified by a genome-wide association study. Cancer research. 2012;72:1182-9.

4. Azzato EM, Tyrer J, Fasching PA, Beckmann MW, Ekici AB, Schulz-Wendtland R, et al. Association between a germline OCA2 polymorphism at chromosome 15q13.1 and estrogen receptor-negative breast cancer survival. Journal of the National Cancer Institute. 2010;102:650-62.
