# Supplementary material for: Prediction of Breast Cancer Survival Using Clinical and Genetic Markers by Tumor Subtypes
Source: PLoS One. 2015 Apr 13;10(4):e0122413. doi: 10.1371/journal.pone.0122413 (PMC4395109; doi:10.1371/journal.pone.0122413)
Supplement: S3 Table — (DOCX) [file pone.0122413.s004.docx]

| S3 Table. Sensitivity analysis on associations between selected SNPs and disease-free survival (DFS) in breast cancer patients by tumor subtypes with stage I-III | | | | | | | | | | | | | | | |
| --- | --- | --- | --- | --- | --- | --- | --- | --- | --- | --- | --- | --- | --- | --- | --- |
| SNP | Loci | Nearby gene | Alleles^a^ | | Discovery set | | | | Replication set | | | | Combined set | | |
|  |  |  |  |  | RAF | HR^b^ | (95% CI) | *P* | MAF | HR^b^ | (95% CI) | *P* | HR^c^ | (95% CI) | *P* |
| **HR+ HER2-** |  |  |  |  |  |  |  |  |  |  |  |  |  |  |  |
| rs161041 | 5q32 | *PPP2R2B* | C | T | 0.14 | 2.17 | (1.55-3.03) | 6.05×10^-6^ | 0.13 | 0.83 | (0.49-1.39) | 0.48 | 1.37 | (0.54-3.51) | 0.51 |
| rs2835688 | 21q22 | *intergenic/DSCR3* | A | G | 0.26 | 2.26 | (1.52-3.37) | 6.23×10^-5^ | 0.27 | 1.06 | (0.73-1.54) | 0.76 | 1.54 | (0.74-3.24) | 0.25 |
| rs9935088 | 16q23 | *WWOX* | A | G | 0.41 | 1.76 | (1.34-2.32) | 4.86×10^-5^ | 0.41 | 0.88 | (0.62-1.24) | 0.57 | 1.25 | (0.64-2.47) | 0.51 |
| rs166870^d^ | 15q25 | *intergenic/MTHFS* | C | T | 0.13 | 2.26 | (1.59-3.20) | 5.58×10^-6^ | 0.13 | 1.95 | (1.01-3.74) | 0.05 | 2.19 | (1.61-2.97) | 5.73×10^-7^ |
| **HR+ HER2+** |  |  |  |  |  |  |  |  |  |  |  |  |  |  |  |
| rs1896346 | 12q24 | *intergenic/TBX3* | G | A | 0.38 | 0.19 | (0.09-0.39) | 7.29×10^-6^ | 0.44 | 1.03 | (0.59-1.82) | 0.92 | 0.45 | (0.09-2.35) | 0.34 |
| rs12940572 | 17q21 | *LASP1* | T | C | 0.29 | 4.11 | (2.14-7.90) | 2.30×10^-5^ | 0.35 | 1.12 | (0.63-1.97) | 0.70 | 2.12 | (0.59-7.59) | 0.25 |
| **HR- HER2+** |  |  |  |  |  |  |  |  |  |  |  |  |  |  |  |
| rs34073156 | 8q24 | *intergenic/LOC100129367* | G | A | 0.10 | 4.67 | (2.56-8.52) | 4.89×10^-7^ | 0.07 | 1.78 | (0.80-3.97) | 0.16 | 3.00 | (1.17-7.69) | 0.02 |
| rs10906761^e^ | 10p13 | *intergenic/CDNF* | C | T | 0.49 | 0.24 | (0.13-0.43) | 3.13×10^-6^ | 0.48 | 0.96 | (0.55-1.68) | 0.89 | 0.48 | (0.12-1.87) | 0.21 |
| **HR- HER2-** |  |  |  |  |  |  |  |  |  |  |  |  |  |  |  |
| rs10825036 | 10q21 | *intergenic/PCDH15* | T | G | 0.32 | 2.44 | (1.68-3.54) | 2.53×10^-6^ | 0.29 | 1.64 | (0.96-2.80) | 0.07 | 2.10 | (1.44-3.06) | 1.18×10^-4^ |
| rs10862597 | 12q21 | *intergenic/RPL6P25* | C | G | 0.32 | 2.11 | (1.36-3.28) | 9.05×10^-4^ | 0.30 | 1.09 | (0.69-1.72) | 0.73 | 1.52 | (0.80-2.91) | 0.20 |
| Abbreviations: SNP, single-nucleotide polymorphism; DFS, disease-free survival; RAF, risk-allele frequency; HR, hazard ratio; CI, confidence interval; MAF, minor allele frequency; HR, hormone receptor; HER2, human epidermal growth factor receptor 2. | | | | | | | | | | | | | | | |
| ^a^Major and minor alleles. | | | | | | | | | | | | | | | |
| ^b^Cox proportional hazard model adjusted for age, recruiting center, and TNM stage. | | | | | | | | | | | | | | | |
| ^c^Random-effect meta-analysis of discovery and replication set. | | | | | | | | | | | | | | | |
| ^d^A proxy SNP, rs1081228, was genotyped for rs166870 in the replication set (r^2^ = 0.96 and D' = 1.00 in CHB+JPT). | | | | | | | | | | | | | | | |
| ^e^A proxy SNP,rs4750561, was genotyped for rs10906761 in the replication set (r^2^ = 1.00 and D' = 1.00 in CHB+JPT). | | | | | | | | | | | | | | | |
|  | | | | | | | | | | | | | | | |
