# Supplementary material for: Prediction of Breast Cancer Survival Using Clinical and Genetic Markers by Tumor Subtypes
Source: PLoS One. 2015 Apr 13;10(4):e0122413. doi: 10.1371/journal.pone.0122413 (PMC4395109; doi:10.1371/journal.pone.0122413)
Supplement: S4 Table — (DOCX) [file pone.0122413.s005.docx]

| S4 Table. Sensitivity analysis on associations between different combined groups of clinical and genetic factors and disease-free survival (DFS) in breast cancer patients subtypes with stage I-III | | | | | | |
| --- | --- | --- | --- | --- | --- | --- |
|  | Discovery set | | | Replication set | | |
|  | HR^a^ | (95% CI) | *P*_Log-rank_ | HR^a^ | (95% CI) | *P*_Log-rank_ |
| Group by RPA among HR+ HER2- breast cancer patients | | | | | | |
| Group 1: TNM stage 0-II and rs166870_CC+CT_ | 1.00 | ref. | 2.61×10^-11^ | 1.00 | ref. | 7.47×10^-08^ |
| Group 2: TNM stage 0-II and rs166870_TT_ | 5.37 | (1.94-14.88) |  | 2.04 | (0.92-4.53) |  |
| Group 3: TNM stage III and rs166870_CC+CT_ | 3.31 | (2.09-5.24) |  | 0.63 | (0.63-14.81) |  |
| Group 4: TNM stage III and rs166870_TT_ | 9.60 | (1.31-70.59) |  | 2.94 | (2.94-17.87) |  |
| Group by RPA among HR- HER2- breast cancer patients | | | | | | |
| Group1: rs10825036_TT+TG_ | 1.00 | ref. | 1.66×10^-04^ | 1.00 | ref. | 2.05×10^-02^ |
| Group2: rs10825036_GG_ | 3.49 | (1.80-6.76) |  | 2.19 | (1.11-4.33) |  |
| Abbreviations: DFS, disease-free survival; HR, hazard ratio; CI, confidence interval; RPA, recursive partitioning analysis; HR, hormone receptor; HER2, human epidermal growth factor receptor 2. | | | | | | |
| ^a^Cox proportional hazard model adjusted for age and recruiting center, and TNM stage. | | | | | | |
|  | | | | | | |
